# A new data-driven paradigm for the study of avian migratory navigation

**DOI:** 10.1186/s40462-025-00543-8

**Published:** 2025-03-11

**Authors:** Urška Demšar, Beate Zein, Jed A. Long

**Affiliations:** 1 Norwegian Institute for Nature Research, Trondheim, Norway; 2 Department of Geography and Environment, Centre for Animals on the Move, Western University, London, ON Canada; 3 School of Geography & Sustainable Development, University of St Andrews, Irvine Building, North Street, St Andrews, KT16 9AL Scotland, UK

**Keywords:** Avian navigation, Multi-modal navigation, Multi-scale navigation, Data-driven methods, Tracking data, Environmental data, Data mining, Machine learning, Artificial intelligence

## Abstract

Avian navigation has fascinated researchers for many years. Yet, despite a vast amount of literature on the topic it remains a mystery how birds are able to find their way across long distances while relying only on cues available locally and reacting to those cues on the fly. Navigation is multi-modal, in that birds may use different cues at different times as a response to environmental conditions they find themselves in. It also operates at different spatial and temporal scales, where different strategies may be used at different parts of the journey. This multi-modal and multi-scale nature of navigation has however been challenging to study, since it would require long-term tracking data along with contemporaneous and co-located information on environmental cues. In this paper we propose a new alternative data-driven paradigm to the study of avian navigation. That is, instead of taking a traditional theory-based approach based on posing a research question and then collecting data to study navigation, we propose a data-driven approach, where large amounts of data, not purposedly collected for a specific question, are analysed to identify as-yet-unknown patterns in behaviour. Current technological developments have led to large data collections of both animal tracking data and environmental data, which are openly available to scientists. These open data, combined with a data-driven exploratory approach using data mining, machine learning and artificial intelligence methods, can support identification of unexpected patterns during migration, and lead to a better understanding of multi-modal navigational decision-making across different spatial and temporal scales.

## Introduction

Migratory birds make journeys that cross oceans, deserts, and mountain ranges, navigating with exceptional skill to their specific breeding and wintering areas. Our understanding of how they do this remains limited, despite decades of research [1]. While there is considerable knowledge about the mechanisms and capabilities birds possess for migration; less is known about what happens in the wild and specifically we do not fully understand how migratory birds use local information from moment to moment to navigate to a distant target. While birds likely use globally available cues, such as the position of the Sun and stars or the strength of Earth’s magnetic field, there is a debate on how they translate this information into movement decisions that accurately lead them to a distant target.

There exists a vast amount of literature in both ecology and neuroscience on the use of various cues and strategies in navigation, however, we note that definitions vary and there are also many contradictory findings [2]. It is not the purpose of this article to review all this literature, but we provide a quick overview of the main developments and terminology.

Navigation is a process of determining and maintaining a course from the origin to a destination regardless of the distance or way of travel [3]. As Able [2] points out, in the past many researchers defined the term “navigation” in many different ways, which led to substantial confusion and therefore it is best to adopt a broad definition of the general term and define specific navigation types separately. We note that navigation is not limited to migration, as similar processes are necessary to move from origin to destination during for example foraging trips, homing or other shorter trips.

The two commonly considered types in the study of avian navigation are vector navigation and true navigation, which we define here (although we note that other navigation types may also occur, such as piloting [4] or path integration, but see the controversy about path integration as described in [2]). Vector navigation is the ability to maintain a specific pre-determined direction for a specified time or distance [5]. This can also be done as a sequence of individual steps in different directions, each lasting for a specified time or distance. This type of navigation is also called a clock and compass orientation strategy and is believed to be used by inexperienced migrants. Birds can determine compass direction through their physiological compass using information from the Sun, the stars, the polarised light or Earth’s magnetic field [6, 7].

The second navigation type is true navigation, which is defined as the ability to navigate to a distant target after displacement to an unfamiliar location, using only locally available cues [8–10]. The implication of this is that the animal needs to have a spatial representation of where it is located relative to the target (positioning) and the sensory information to orient itself (determining the compass direction) that can be extrapolated beyond the local range [5]. The combination of positioning and orienting is traditionally described as a “map and compass” strategy [11]. There is general consensus that orienting and maintaining direction in flight is done by a time-compensated Sun compass, by a time independent stellar compass or by a magnetic compass when the Sun or stars are not visible [6, 7]. However, the sensory basis for the map has not been determined and is subject to much debate [12]. In neuroscience, such a spatial representation is called a cognitive map, which is a mental representation of geographic reality [13, 14]. For humans, a cognitive map consists of knowing positions and spatial relations between landmarks in the external world [15]. For birds and other migratory animals, the cognitive map, if it exists, may involve more than just landmarks, and positioning information may come from visual, geomagnetic, olfactory and acoustic sources [12].

Two potential types of a cognitive map have been proposed for birds: a mosaic map and a bi-gradient map. A mosaic map [2] is the equivalent of the human cognitive map, in that it is formed from learned spatial relationships between landscape features and key locations (e.g. in human terms home, school or work). It has been proposed that these landscape features can either be visual landmarks or odours that guide olfactory navigation (see this review [2] for original references for both suggestions). There has been contradictory evidence to existence of either of these two mosaic map types for birds and other animals and specifically no GPS-based studies have been able to confirm its existence for birds. We note that a recent study using high spatial and temporal resolution GPS data found evidence that is consistent with use of a landmark-based cognitive map by wild bats during foraging [16]. Of note however is that a map of this type would by its nature only be useful over the geographical extent of the learning, which may be of limited application for long-distance navigation over unfamiliar terrain.

The second type of proposed map is a gradient map or a grid map, where at least two cues vary across large regions in a systematic and stable manner, to facilitate positioning in a similar way as humans use the two Cartesian dimensions (i.e. X and Y) on a map [2]. Bi-gradient maps could theoretically be based on geomagnetic information [6]: magnetic inclination and intensity generally vary across the North-South axis and could be used as determination of latitude. In some parts of the world declination varies in the East-West direction and could be used to determine longitude. However, there is to date no empirical evidence from tracking data that a geomagnetic map exists (e.g. see [17]). In our recent work based on GPS tracking and contemporaneous real geomagnetic data we also found such a strategy highly unlikely [18, 19]).

For olfactory navigation, the map does not have the structure of a bi-coordinate system, which would allow determination of the exact position and distance to the goal, but instead provides information only about the direction to the goal [20]. The olfactory map hypothesis states that birds are assumed to learn windborne odours associated with wind directions at home. When displaced, they find the direction in which they need to fly based on local concentrations of odours. It has been shown that volatile organic compounds in the atmosphere are distributed along stable spatial gradients, and that their ratios would provide sufficient information for the olfactory map, even for long-distance navigation, such as during migration [21]. This type of map is fairly established for homing pigeons [20], but wild birds might use different strategies [17]. For example, migratory birds might follow an odour plume located along a migration corridor [22]. Pelagic seabirds that need to navigate between colonies and distant foraging grounds may learn the olfactory topography of the ocean, i.e. the landscape of patches of odours originating from islands, coasts and areas rich with plankton [17].

Less research has been done on acoustic navigation, perhaps because of lack of possibilities to obtain suitable data on environmental infrasound. Theoretically, acoustic sources could support piloting (moving between acoustic landmarks), beaconing (following a gradient to a source) or a bi-gradient map [23]. None of this has been confirmed, although there are first indications that infrasound might be used by seabirds as a navigational cue [24].

For more information on terminology and state of the art on avian navigation see review papers on definitions [2], compass orientation [7], positioning [12], mechanisms and cross-species comparisons [5, 25], geomagnetic navigation [6], olfactory navigation [17, 20] and acoustic navigation [23]. We further note that, unlike in neuroscience, in avian navigation literature, the term “navigation” often refers only to true navigation. Vector navigation or other types of navigation (e.g. piloting– moving between landmarks, beaconing– following a gradient to a source, path integration– keeping track of direction and distance travelled, etc [15]). are sometimes not included. In this paper we use the term navigation in the broadest sense, as defined earlier, i.e. as the process of determining and maintaining a course from the origin to a destination. This can include any type of mechanism that supports this process and is not limited to true navigation.

Traditionally, ethologists have studied bird navigation with laboratory behavioural experiments [26], on their own or in a combination with displacement experiments [27, 28], where researchers observe how captive birds change their departure direction when cues are artificially changed. Recent studies use tracking data, collected with in-situ locational devices placed on wild migrants, such as GPS trackers [29, 30], radiotelemetry [31] or geolocators [32]. Tracking has provided insights on where and when wild birds migrate and has also been used for study and comparison of various types of navigation strategies. See for example Wikelski et al. [33] who combined tracking and displacement to compare olfactory and geomagnetic navigation in migrating gulls. There are many other studies using a combination of displacements and tracking, here we only list a few examples. Two studies look at migration of young and adult common cuckoos [34, 35] and another one compares olfactory and landmark-based navigation in pelagic seabirds [36].

There is also substantial literature that focuses on physiological capabilities of how birds sense various navigational cues. This includes the search for the receptor that could sense geomagnetic field, with three proposed options: the photoreceptor radical pair hypothesis [37], the magnetite-based sensor [38] and a possibility of using electromagnetic induction to sense the field [39]. We note that the three proposed mechanisms are not necessarily mutually exclusive.

Navigational strategies may vary during the journey depending on encountered conditions, as birds take advantage of the cue that is most available at different times during their journey [5]. For example, the lack of visible landmarks during the night or overcast conditions that prevents the birds to see the Sun or stars may lead towards increased use of geomagnetic cues [6]. Bingman and Cheng [5] propose a framework where different sensory cues and representations are used at different spatial scales, resulting in a multi-modal navigation decision-making process, where birds continuously switch between different modalities. The unsolved question is how and when the bird changes from one mode to another and how/if the switch is related to the encountered environmental conditions.

Additionally, navigation decisions need to be made across different spatial and temporal scales. Mouritsen [6] proposes that long-distance navigation operates across three scales: a long-distance phase, a narrowing-in or homing phase and a pinpointing-the-goal phase. The long-distance phase involves navigation far away from the home range and relies on global cues, such as celestial or geomagnetic information. This phase prioritises compass-based orientation either through vector navigation (for inexperienced migrants) or by adjusting compass headings based on learnt gradient maps. Narrowing-in or homing requires local learned maps of a variety of senses and environmental cues within the home range, such as local olfactory landscape. Finally, the pinpointing-the-goal phase relies on visual or olfactory landmarks close to the destination location. The process of switching between global and local phases may repeat itself many times during the journey, for example when stopovers serve as intermediate targets [40]. Birds also adjust and re-orient during the journey from time to time (see for example [41–43]). Further, navigation at both global and local scales may be affected by local atmospheric conditions, such as wind and uplift [44–46] or by high air pollution [47].

We should therefore consider navigation as a multi-scale and multi-modal process, that may change during the course of migration to leverage different available cues, react to environmental conditions, or switch between coarse and fine-scale navigation strategies. This is however challenging to study, because these kinds of questions can only be answered with long-term (life-long) tracking data on wild migrants, along with co-located and contemporaneous environmental data [40, 48]. Leveraging these kinds of data is difficult and requires a new methodological approach that will identify patterns of navigational decision-making from combined data.

**Fig. 1:**
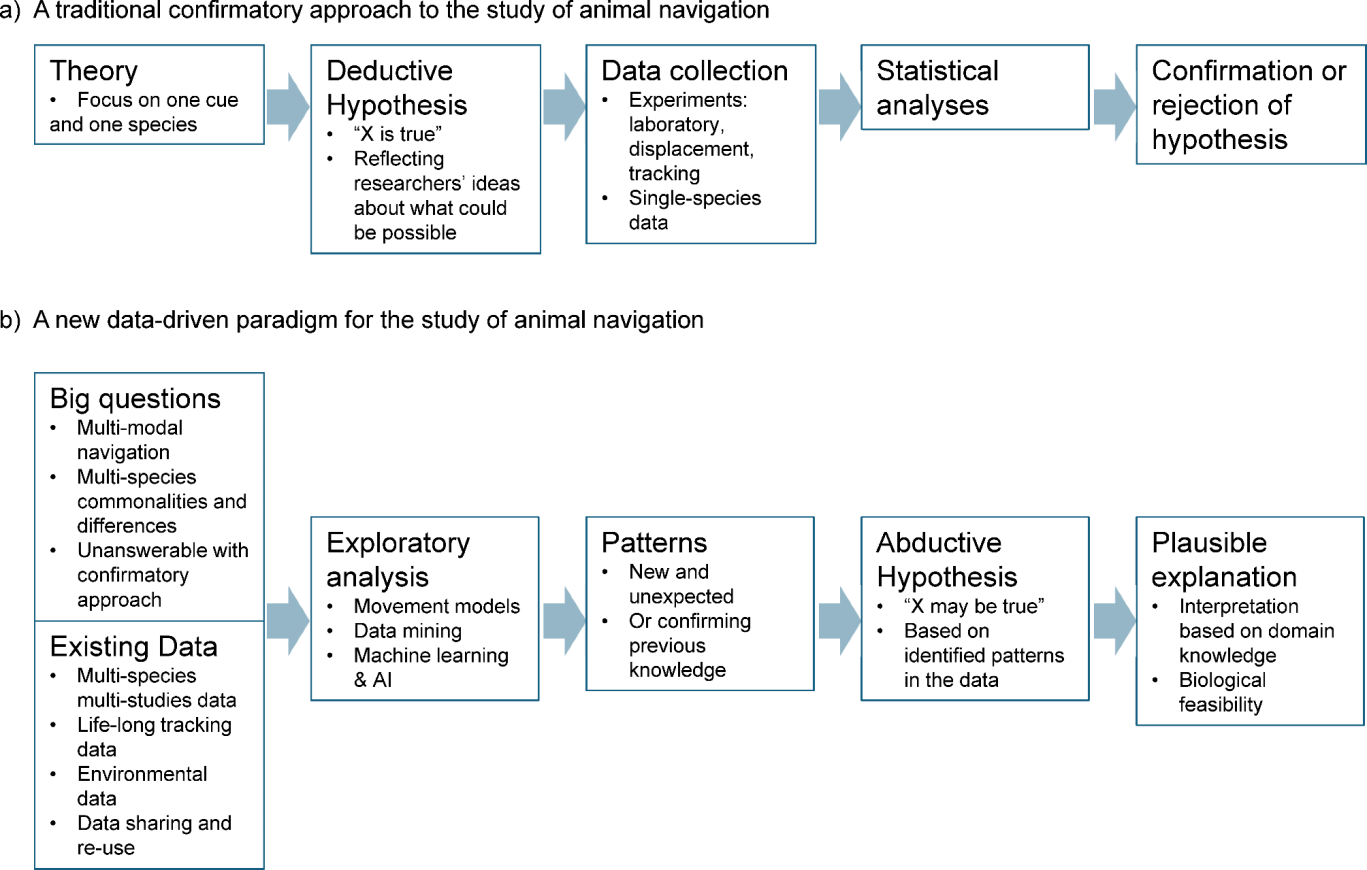
The traditional theory-driven confirmatory approach vs. the new data-driven abductive reasoning paradigm

In this paper we propose that a new data-driven approach, based on exploratory analysis of existing long-term tracking data and co-located and contemporaneous environmental data, can support the study of avian navigation as a multi-scale and multi-modal process. A data-driven approach is increasingly common in scientific disciplines which have become data-rich [49, 50], and complements a traditional theory-driven approach. A theory-driven approach is based on deductive reasoning process where a hypothesis is set first (i.e. “X is true”) and then data are collected to either confirm or reject the hypothesis. That is, the research question comes first and then data are collected to address this question (and only this question). In a data-driven approach, we start with a large amount of data that already exist and develop methods to identify patterns in the data (Fig. 1). This reverses the process, as we start with data that are collected widely and at large scales without a particular research question in mind, and then these data are analysed to come up with a research question. Consider for example mobile phone data: these are collected continuously by mobile phone providers for millions of users, but can then be used in a secondary analysis to study human mobility [51]. An analogy to mobile phone data in movement ecology are life-long tracking data of large groups of individuals, which are streamed live to an online portal, such as Movebank [52], ready for secondary analysis. Typical methods for a data-driven analysis include data mining [53], machine learning [54] and artificial intelligence (AI) [55]. Identified patterns are used to form a so-called abductive hypothesis, which states that “X may be true”. This hypothesis can be interpreted into a plausible explanation using domain knowledge. For more information on the distinction between how the theory-driven (question first) and data-driven (data first) approaches work in practice see the Integrated Bio-Logging Framework [56].

Following trends in other fields, ecology as a discipline is now also starting to take advantage of data-driven methods [57]. However, many ecology researchers continue to prefer more traditional confirmatory statistical modelling [56] and uptake of data-driven methods such as machine learning is slow [58]. In terms of navigation, there are to date only a few data-driven studies, especially of long-distance migrants. We list these studies in the second part of this paper.

We propose that, due to the recent influx of high-resolution animal tracking data and readily available suite of environmental data (largely from satellite remote sensing), it is now time to employ data-driven approaches for the study of avian navigation. In the rest of this paper, we explain how this could be done: we first discuss which data needed for study of navigation exist, then consider data-driven methods and finally emphasise the importance of interdisciplinary collaborations in this endeavour.

## Navigational Umwelt

**Fig. 2:**
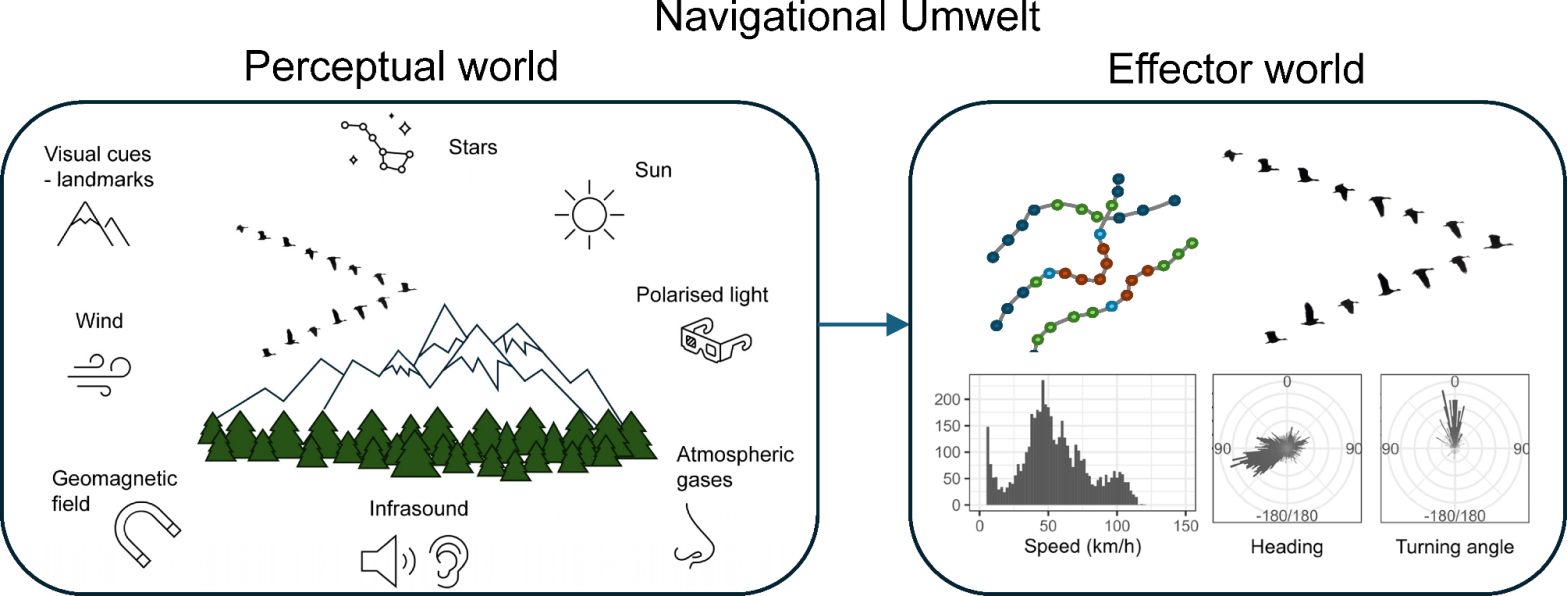
Navigational Umwelt of a migrating individual consists of its perceptual world and effector world. Perceptual world is full of potential navigational cues and environmental factors (e.g. wind). The individual’s response to the experienced values of these cues and factors leads to navigational decisions. These decisions are reflected in movement patterns as part of the effector world. In data they are represented as geographic trajectories and related movement parameters (speed, heading, turning angle and others)

In early 20th century, a Baltic-German biologist Jakob von Uexküll introduced the idea of an Umwelt [59], to conceptualise the problem of how living beings perceive their environment and how this perception determines their behaviour. The Umwelt (a German word for environment) consists of the perceptual world, which is the part of the environment that an animal is able to sense, and the effector world, which is its behaviour as a response to the environmental conditions. Every species has its own unique Umwelt that is not accessible to other species. The Umwelt of each individual also constantly changes. Navigational Umwelt (Fig. 2) includes the actual environmental conditions that the individual experiences during migration, along with the navigational decisions based on these conditions, its internal state, motion, and navigation capacity [60]. These navigational decisions lead to movement patterns, which can be described in terms of trajectories and movement parameters.

## Sensing the Umwelt: data sources and data fusion for navigational Umwelt

Recent technological advances now allow researchers to collect data on both the perceptual world (environmental data) and effector world (GPS tracking data). To understand what the animal perceives and how it reacts at each moment, we need both long-term movement data on wild migrants, as well as co-located and contemporaneous environmental data. Here we review sources of data needed to study both perceptual and effector worlds.

Long-term movement data are collected widely, as improved tracking technologies now support life-tracking at increasingly detailed spatial and temporal scales [61, 62]. Development of re-chargeable trackers with solar panels means that many of these data are live-streaming from individuals for years, resulting in a rich resource for study of movement behaviour. These data are stored in standardised form on portals such as Movebank [52], which, at the time of writing, contains over 6.3 billion animal location data points from over 1400 species. The vast majority of Movebank data are either openly available or available upon request for secondary data re-use, supporting collaborations among ecologists across species and geographies. This has led to significant advances in movement ecology, such as studying movement responses to climate and environmental change in the Arctic [63], or discovering how human activity during COVID-19 pandemic influenced terrestrial mammals across the globe [64]. However, to date, no such collaborative multi-species and geographically spread studies exist for avian navigation.

Environmental data are widely available from direct observations (such as weather data), remote sensing and various other geophysical data collections. As governmental agencies increasingly adopt open data policies, their data are freely available online, for example weather data from the European Centre for Medium-Range Weather Forecasts (ECMWF) or from national meteorological agencies. Space agencies, including European Space Agency (ESA) and the National Aeronautics and Space Administration (NASA) also provide satellite data openly. Further potentially relevant data for the study of navigation, such as astronomical data on positions of the Sun and stars are available from open-source astronomical software packages. Table 1 provides a summary of open data for navigational cues and environmental conditions.

**Table 1:** A non-comprehensive selection of open environmental data for navigational cues and environmental variables

Cue or variable	Data description	Example data sources
Sun and stars	Astronomical software packages	There are many, but we give some examples: R packages suncalc [65] or suntools [66] for position of the Sun and moon; Python library AstroPy [67] for Sun and star calculations
Visual landmarks	GIS and other spatial data on geographical features, points of interest etc.	There are many sources, from global data bases (OpenStreetMap (OSM) [68], Natural Earth [69]) to national sources (Digimap [70] in UK)
Earth’s magnetic field	Terrestrial measurements	International Real-Time Magnetic Observatory Network INTERMAGNET [71]
Earth’s magnetic field	Satellite measurements	Data from ESA Swarm constellation [72]
Atmospheric composition	Terrestrial measurements	Data from national air pollution networks, e.g. UK Air [73]; global WHO Air quality database [74]
Atmospheric composition	Satellite measurements	Data from Sentinel-5P [75], Aura [76], MISR instrument on Terra satellite [77], MAIA satellite [78]
Environmental infrasound	Data on wave height, used to model natural infrasound	Satellite data from Sentinel-3 [79]; wave height data from ECMWF ERA5 reanalysis [80]
Environmental infrasound	Data on anthropogenic infrasound	Seismic noise data from the International Federation of Digital Seismometer Networks [81]; human mobility data for road traffic noise [82]
Wind and weather	Meteorological and climate data	ECMWF ERA5 reanalysis data of the hourly global climate conditions [80]; data from national meteorological agencies, e.g. UK MetOffice data [83]

Environmental data for sensing navigational Umwelt therefore exist. What is missing are methods to link these data together across space and time in a way represent environmental variables at the scale at which movement data are collected. This process is called spatio-temporal data fusion. Movement ecology tools for fusion of movement and standard remotely sensed and meteorological data exist (e.g. the Env-Data system as part of Movebank [84]), as well as methods for multi-spectral optical and radar remote sensing data [85, 86]. Further, many of these datasets are now available through online platforms such as Google Earth Engine and can be readily linked with animal tracking data [87].

However, study of avian navigation requires information on global navigational cues that are not sensed with standard optical and radar sensors, such as Earth’s magnetic field, atmospheric composition and environmental infrasound. Tools for linking movement data with these alternative data do not currently exist, with one exception, our MagGeo tool that links satellite data on Earth’s magnetic field to animal tracking data [88]. The reason for this is that these data come in unusual formats, specific to what satellites are able to observe from the orbit. For example, for satellite data on concentrations of atmospheric pollutants (e.g. NO_X_, SO_2_), instead of presenting reflectance of the Earth’s surface, each pixel represents the total aerial column of a pollutant, from the ground to the top of the atmosphere. This is therefore an indirect measure of the ground concentration of each gas and needs to be translated to the ground value using modelling approaches [89]. Linking such complex environmental data to movement data therefore requires substantial expertise in both spatio-temporal data fusion and the domain for which they were originally collected (e.g. geomagnetism, atmospheric chemistry, environmental acoustics).

## Studying navigational decision-making: a data-driven analysis

Once appropriate data have been identified and joined, the next step in a data-driven approach is the exploratory analysis to interrogate the data and identify patterns (Fig. 1). Here we outline four areas of opportunity for employing data-driven methods in the field of avian navigation, starting from recent developments towards more speculative ideas.

### Individual-based models and simulations

Some recent navigational studies already employ a data-driven approach with statistical models, but formulate them in a way to focus on navigation. The main model type are agent-based models, where navigational behaviour of individuals is simulated based on movement properties and environmental conditions. Examples include individual-based models [90, 91] and correlated biased random walks [18, 19] for geomagnetic navigation. Some olfactory navigation studies use particle and atmospheric models for VOC distributions to create simulations [22, 92].

When simulations assume that environmental conditions do not vary across time, this can introduce bias and uncertainty in the model. An example are studies of geomagnetic navigation which employ a static model of the Earth’s magnetic field [90, 91]. This is a problem, because the geomagnetic field changes across space and over time under the influence of solar wind. When geomagnetic field is highly disturbed, such as during geomagnetic storms, this may affect migrating animals. For example, we found that greater white-fronted geese have an increased speed and a wider range of turning angles during geomagnetic storms [88]. This finding is supported by historical observations of bird’s reactions to sudden changes in geomagnetic activity [93, 94], but given the rarity of large geomagnetic storms and inability to observe birds during these storms, this is not something that could be studied in detail previously. Considering the field dynamics and using correct temporally changing values of the field in the individual-based models is therefore crucial as we have recently shown [18, 19].

### Spatial optimisation models

Multi-modality of navigation could be defined as a spatial optimisation problem. In contemporary engineering and technology, numerous difficult problems, which are typically non-linear and complex, are defined and solved as optimisation problems. An optimisation problem has three parts: an objective function which needs to be optimised, constraints under which this optimisation needs to happen, and decision variables [95]. When the problem involves decision-making using geographic information, this can be termed spatial optimisation [96]. A spatial optimisation algorithm maximises or minimises an objective related to a geographic phenomenon, which involves defining a suitable objective function with specific spatial constraints. In terms of navigation, the objective function could be a route to the target destination under specific environmental conditions while minimising navigational errors.

Optimisation algorithms in engineering and technology are often defined based on animal movement behaviour– Amiri et al. [95] list over fifty methods that have been developed based on movement behaviour of diverse variety of mobile organisms, from ants to foxes, gorillas, orcas, prairie dogs, moths, hummingbirds, cuckoos and so on. However, the opposite knowledge transfer from engineering to ecology seems to be lacking, as there are very few studies that utilise optimisation as methodology to study animal movement. For animal navigation in particular, optimisation algorithms are virtually untested, with exception of one study, which uses an evolutionary optimisation algorithm to explore a long-term change in geomagnetic navigation strategies [97].

Spatial optimisation algorithms have been applied to a variety of routing problems in robotics [98], vehicle navigation [99] and prediction of human movement behaviour [100]. For example, a study in social robotics [101] plans trajectories of assistive robots in a situation where these robots have to find the optimal path through a crowd of people. They propose to use a spatial optimisation method, the inverse reinforcement learning (IRL), for this purpose. IRL is the problem of finding latent preferences in decision-making from observed sequential decision-making behaviour. That is, we assume that the behaviour of an agent is optimal and try to identify the preferences that led to this behaviour. In the case of social robots [101], the IRL algorithm learns the socially adaptive behaviour of moving through crowds based on environmental factors (density and velocity of pedestrians). The resulting model is then used to generate the optimal route for each robot. Such approaches could be applied in the bird navigation context using bird GPS trajectories and data on contemporaneous and co-located environmental conditions. Training an IRL model on these data could help identify the unknown preferences that would represent how a migrating bird uses environmental conditions to follow the optimal route to the target destination.

### Data mining

Since the goal of the data-driven analysis is to find navigation-related patterns; data mining can facilitate the exploration of navigational multi-modality. Data mining is the process of identifying as-yet-unknown patterns and knowledge in data [53] and has been used in many disciplines for decades. For example, most analysis tasks for optical and radar remote sensing data are done with data mining methods, such as creating land use maps or segmenting images or videos into objects. Traditional data mining methods include methods for clustering, classification and association [53]. Clustering identifies groups of data points with similar properties, classification assigns a class label to each data point based on a previously known model of grouping and association identifies data points which are commonly found near each other in the attribute space.

Clustering is used in movement analysis for identification of different behaviours as a response to environmental conditions, specifically in cases where the number and type of these behaviours is initially unknown. Clustering algorithm is run on either movement parameters (speed, acceleration), or environmental data (weather data), or both together. Here we give an example from human mobility: Brum Bastos et al. [102] use clustering of GPS trajectories of human commuters to identify which individuals respond similarly to similar environmental conditions. They run hierarchical clustering on a set of environmental conditions (thermal comfort, wind direction and speed, temperature, humidity, daylight, rainfall) to identify which trips are similar to each other. Trips are grouped into clusters, which are explored in terms of travel mode (e.g. walking, taking the car), to identify if the choice of mode corresponds to environmental conditions.

Contrary to clustering, which identifies groups of similar objects with no pre-conception of what those groups are, classification groups data points into known classes. This means that, when used for analysis of movement behaviour, the number and type of behaviours need to be known a-priori. An example study from movement ecology [103] builds classification trees from GPS and accelerometer data. The algorithm checks a number of predictor variables against given behaviours (e.g. in this case bird movement behaviours: no movement, flying, terrestrial movement) and develops a data mining model (a classification tree), where data points with specific combinations of values in predictor variables are classified as having a specific behaviour.

Association rules are less commonly used in movement data mining, but there is an example study [104], which uses this method to link animal behaviours to environmental factors, with a focus on presence of other animals. They first segment GPS trajectories into individual behaviours and then build association rules that link each behaviour to geographic distance to another animal.

For more info on data mining methods in analysis of movement see our interdisciplinary reviews on animal and human movement analysis [51, 105].

In terms of navigation, one question which data mining might resolve is to identify when birds decide to switch between different navigational modalities and how the timing of these decisions relates to specific environmental conditions. Bingman and Cheng [5] propose two possible control processes on how this might work. One is that navigational strategies are employed sequentially and triggered by specific conditions. Another one is that all strategies operate in parallel throughout the journey, but that one (or several) dominate at different times. It is unclear which of the two processes operates at physiological level, but switching between several strategies may lead to better navigational accuracy as a consequence of using multiple cues. Our on-going work uses hierarchical clustering, a common data mining approach, to explore this process. We cluster GPS points annotated with geomagnetic, meteorological, geographical and movement variables to find groups of points with similar movement behaviour. The goal is to identify which cues and strategies dominate the flights at different points in time, how this differs among individuals and when these strategies switch from one to another. As far as we are aware, this is the first attempt to use clustering for a study of avian navigation, in spite of this being a relatively simple and frequently used data mining method in analysis of movement.

### Machine learning and AI models

A subset of data mining methods are machine learning models, which can learn from data. That is, as they repeatedly analyse the data during the so-called training process, they have the ability to adjust their own parameters. This adjustment process means that a model that has trained itself without external instruction to recognise patterns in the data and is able to generalise these patterns to unseen data [54].

There are many machine learning models serving different purposes, but recently a large focus has been on AI models, such as artificial neural networks [55]. Neural networks are a machine learning method inspired by the biological neural networks. They consist of artificial neurons, which are processing units that take weighted input from several sources. The input is transformed into an output using the activation function (in parallel to a biological neuron being activated by electrical signals). A neural network consists of many layers of these artificial neurons, which are connected with each other in various ways, passing information from one to another not just in one direction, but also backwards and across the layers. If the number of layers is large, the network is called a deep network and the algorithm a deep learning algorithm [53]. Additionally, deep learning algorithms take all the available raw data and automatically determine which attributes are relevant for analysis (this is called automated feature extraction).

After the network architecture is defined (i.e. the number of neurons and layers and the structure of connections), it is then trained on data. A training data set is repeatedly fed into the network, which continuously adjusts the weights of each neuron (this is the “learning” in machine learning) and returns outputs. This process continues until the output and the parameters stop changing. A trained network can then be used on similar data to predict the outputs– for example, in a Large Language Model, such as ChatGPT (which is essentially a large and very complicated deep neural network built for generating text), the training data is all the text on the internet, the data to be analysed is the question that the user poses and the output is artificial text that is statistically similar to a related pattern in the training data.

There are different types of neural networks solve specific problems. Image processing uses convolutional neural networks (CNNs) [53]], which take images as input and decompose each image into smaller subsections (one into each neuron) and apply filters to each subsection. Each sub-section results are then re-combined using convolution to build a new representation of the image. This process can be used to recognise objectsin the image data. CNNs have found their way into movement ecology problems which require handling large data sets of images, such as identifying individuals from camera trap images [106] and videos [107]. Another example are CNNs that use drone-recorded videos to automatically track the location and body posture of free-roaming animals in a 3D landscape [108].

Another type of neural networks in movement ecology are deep learning models for analysis of trajectories. They are used for comparative analysis of trajectory segments [109] to distinguish characteristic movement patterns of indviduals. They are also used to infer behavioural patterns from simpler movement data, for example diving behaviour from GPS trajectories [110] or dynamic body acceleration from depth data [111]. This solves the problem of having to fit costly additional sensors, such as a time-depth recorders or a high-resolution accelerometer.

We are not aware of any applications of neural networks in navigation studies, but here we propose some ideas of how this could be done based on state of the art developments of these models in analysis of human movement.

In human mobility, deep learning is commonly used for both trajectory generation and next-location prediction. Next-location prediction [112] identifies spatial and temporal patterns in historical mobility data, along with social and environmental factors influencing decisions that led to these patterns. These methods, particularly deep learning, capture long-range temporal and spatial dependencies and could model the complex navigational decision-making of migratory birds. Trajectory generation [112] creates simulated trajectories that statistically replicate key mobility features (distance, speed). This is typically used for simulating urban mobility under hypothetical future scenarios, including introduction of new infrastructure, natural disasters, or epidemics. Generative deep learning models [112, 113] can capture the non-linearity and chaotic nature of movement decision-making, and create realistic trajectories. In terms of avian navigation, these models could be used to forecast migration routes under specific environmental conditions.

One reason that machine learning and AI methods are not yet employed for the study of navigation may be the size of training data. AI models require large data sets to be trained. Collecting animal tracking data on a large scale is still more costly and difficult than tracking humans [51] and no single navigation study may be able to generate sufficient data for AI use. Instead, what is needed is data sharing within large collaborations, with interdisciplinary involvement of both ecologists and data scientists. This type of collaboration is however yet to be realized within navigational studies. There are precedents for such collaborations in movement ecology, for example, during COVID-19 pandemic, the International Bio-logging Society (IBS) issued a call to scientists to contribute data from their on-going studies to explore the effect of the decrease in human activity on wildlife [114]. Hundreds of researchers from across the globe answered the call and this has led to a number of multi-species multi-study analyses [64, 115] that would not have been possible otherwise. Recently, the IBS has formalised widespread global data integration through proposing standards and public archiving of bio-logging data [116]. There is an opportunity for navigation researchers to do the same.

Bringing together researchers from ecology and data science and re-using tracking data from many studies would allow for comprehensive data-driven multi-species analyses, not just for birds but also for other migratory species, such as marine animals (turtles [117], whales [118], seals [119]), terrestrial mammals [120], bats [121], fish [122] and insects [123]. Using AI and other data-driven methods on these massive, linked datasets would allow a holistic study of animal navigation in the wild; exploring similarities and differences between species and leading to a better understanding of the incredible ability of long-distance navigation.

## Conclusions

We live in a world that is increasingly data rich and where many things are sensed. These data offer an unprecedented opportunity to better understand our world and its living beings. Many scientific disciplines have already started taking advantage of these vast data resources and have embraced new methodological approaches that look for patterns and knowledge in the data. It is now time for research on avian navigation (and navigation of other species) to do the same. In this paper we have shown that data needed for this endeavour already exist and are widely available. We have outlined four areas that provide exciting possibilities for how a data-driven approach with these data could expand the study of complexities of animal navigation beyond its current limitations. This is a call to the navigation community to start participating in the data-driven revolution and discover what data can reveal about the amazing navigational ability of migrating animals.

## Data Availability

No datasets were generated or analysed during the current study.
